# Hypertonic saline for prevention of delirium in geriatric patients who underwent hip surgery

**DOI:** 10.1186/s12974-017-0999-y

**Published:** 2017-11-14

**Authors:** Xi Xin, Fei Xin, Xuguang Chen, Qi Zhang, Yanan Li, Shuping Huo, Chongfu Chang, Qiujun Wang

**Affiliations:** 1 Department of Anesthesiology, The Third Hospital of Hebei Medical University, No 139, Ziqiang Road, Shijiazhuang, 050051 Hebei People’s Republic of China; 2 Department of Respiration, Tianjin Institute of Respiratory Diseases, Tianjin Haihe Hospital, Tianjin Medical University, Tianjin, 300350 People’s Republic of China

**Keywords:** Hypertonic saline, Postoperative delirium, Elderly, Monocytes, Cytokines, Neuroinflammation

## Abstract

**Background:**

Postoperative delirium (POD) is a common disorder in the elderly patients, and neuroinflammation is the possible underlying mechanism. This study is designed to determine whether or not hypertonic saline (HS) pre-injection can alleviate POD in aged patients.

**Methods:**

This prospective study recruited 120 geriatric patients who underwent hip surgery. The patients were randomly divided into two groups: control group (NS group) and HS group. Patients in the NS group were pre-injected with 4 mL/kg isotonic saline, and those in the HS group were pre-injected with 4 mL/kg 7.5% HS. All 120 patients were then subjected to general anesthesia. Blood samples were extracted to detect the concentration of inflammatory factors, namely, IL-1β, IL-6, IL-10, and TNF-α, and the nerve injury factor S100β. Flow cytometry was used to detect the number of monocytes in peripheral venous blood and evaluate the relationship of inflammation to delirium. The nursing delirium screening scale (Nu-DESC) was used to determine cognitive function 1 to 3 days postoperatively.

**Results:**

Analysis using random-effect multivariable logistic regression indicated that HS administration before anesthesia was associated with a low risk of POD (odds ratio [OR], 0.13; 95% CI, 0.04 to 0.41; *P* = 0.001) and few CD14 + CD16+ monocytes (β = − 0.61; 95% CI, − 0.74 to − 0.48; *P* = 0.000) the following day. When the association between HS and delirium was controlled for CD14 + CD16+ monocytes, the effect size became nonsignificant (odds ratio [OR], 0.86; 95% CI, 0.14 to 5.33; *P* = 0.874). TNF-α was significantly associated with POD (odds ratio [OR], 1.10; 95% CI, 1.05 to 1.16; *P* = 0.000). However, IL-1β, IL-6, IL-10, and S100β were not significantly related to POD.

**Conclusion:**

HS can alleviate POD in geriatric patients and may inhibit the secretion of inflammatory factors by monocytes.

## Background

Postoperative delirium (POD) is a common acute cerebral disorder that is characterized by sudden and transient disturbances in awareness, consciousness, and cognition [1, 2]. POD is an age-related syndrome, primarily occurring in the elderly population (> 65 years of age) and may be associated with increased morbidity, length of hospital stay, and patient care costs [3]. The highest incidence of POD is found in patients with hip fractures (35–65%) particularly 3 days after the operation [4], since the fracture itself and subsequent surgery are two events leading to a systemic response. Elderly patients with preoperative cognitive impairment, dementia, anxiety, depression, and malnutrition are at great risk of POD [5]. The pathophysiology of POD has not been completely understood, and no specific treatments are currently available.

Neuroinflammation plays an important role in the development of delirium [6, 7]. Aseptic surgery increases in inflammatory markers in serum and causes inflammation-mediated, hippocampal-dependent, and cognitive dysfunctions. Increased serum levels of inflammatory cytokines postoperatively are associated with memory impairment, reactive microgliosis, and upregulated interleukin (IL)-1β expression in the hippocampus [6]. Inflammatory cytokines are mainly involved in postoperative cognitive delirium [8]. Cytokines are categorized as inflammatory cytokines, including IL-1β, IL-6, IL-8, and tumor necrosis factor alpha (TNF-α), and anti-inflammatory cytokines, such as IL-10. Inflammatory cytokines may lead to the occurrence and development of diseases, that is, high levels of inflammatory cytokines are related to the severity of the disease [9, 10]. The role of systemic TNF-α has been widely investigated; a high level of systemic TNF-α is associated with twofold increase in disease symptoms, including apathy, anxiety, depression, and agitation [11]. CD14 + CD16+ monocytes exhibit enhanced capacity for TNF-α generation [12]. Thus, restraining and regulating inflammatory factors may improve the prognosis of delirium.

Hypertonic saline (HS) is widely used as resuscitation fluid in patients with traumatic hemorrhagic shock. HS exerts not only beneficial effects on hemodynamic parameters but also modulatory effects on various immune cell functions, such as degranulation, adhesion, cytokine expression, and reactive oxygen species production [13]. HS modulates local and systemic inflammatory response [14, 15], but its effect on alleviating postoperative delirium remains to be discussed.

This study aims to investigate the effect of HS on elderly patients with POD and preliminarily determine the relationship among HS, neuroinflammation, and POD. Results may provide reference for clinical applications. We tested the hypothesis that prophylactic HS could alleviate delirium and could be related to reduction of systemic inflammation.

## Methods

### Patients and setting

This prospective cohort study recruited patients (> 65 years old) who underwent hip arthroplasty for femoral neck fracture surgery in the Third Hospital of Hebei Medical University in July 2017. We collected basic patient information, including age, sex, body mass index (BMI), American Society of Anesthesiologists (ASA) score, coronary artery disease, hypertension, diabetes, and cerebral infarction. The mini-mental state examination (MMSE) was administered to screen for preoperative cognitive function 1 day before the surgery. The exclusion criteria were as follows: ASA physical status of above III, age of 65 and younger, MMSE score of less than 24 or dementia due to various etiologies, preoperative delirium, history of neurological or mental illness, current use of tranquilizers or antidepressants, history of an endocrine or metabolic disorder, recent use of glucocorticoids or other hormones, suffering from infections or chronic inflammatory conditions, intake of anti-inflammatory drugs, unwillingness to complete the experimental procedures, presence of a language barrier, severe hearing or visual impairment, illiteracy, and alcohol or drug dependence. Only 120 patients were enrolled in the study. The patients were divided into two groups (*n* = 60) by using a random number table: 7.5% HS group and normal saline group (NS group). All patients were required to provide written informed consent before randomization. If acute heart failure, renal failure, and electrolyte disorder occurred during the treatment, then the experiment was terminated and relevant measures were performed.

### Anesthesia and surgical management

Invasive blood pressure, pulse oxygen saturation, electrocardiogram, bispectral index, and end-tidal CO_2_ (ETCO_2_) were continuously monitored in the two groups during the perioperative period. In brief, 4 mL/kg 7.5% HS (Shijiazhuang No. 4 Pharmaceutical Co., Ltd.) or NS was injected 30 min before administering anesthesia.

Intraoperative circulatory system was maintained by adjusting the anesthesia depth (controlling BIS 40 to 60) and administering transfusions or cardiovascular agents as necessary. Blood pressure fluctuation amplitude did not exceed 20% of the base value. Scopolamine and penehyclidine were prohibited. Atropine was used only to reverse bradycardia. All patients’ surgery time were controlled within hours. After the surgery, all of the patients were transferred to surgical intensive care unit and received the same treatment.

### Blood sample collection and sample bank establishment

Venous blood was drawn at 06:00 on the first day after the surgery. Blood samples were immediately placed into sterile EDTA test tubes and centrifuged at 3000*g* for 30 min at 4 °C to collect plasma. Plasma was aliquoted into polypropylene tubes and stored in a freezer at − 80 °C (Sanyo, Japan) until further assayed. Additional venous blood was collected for flow cytometry to determine the number of monocytes.

### Cytokine measurement

The concentrations of IL-1β, IL-6, TNF-α (pro-inflammatory cytokines), IL-10 (anti-inflammatory cytokine), and S100β in plasma were quantified with a commercial enzyme-linked immunosorbent assay (ELISA) kit (Duoset, R&D Systems, UK) in accordance with the manufacturer’s instructions. ELISA assays were read by SoftMax Pro version 5.3. All samples were assayed in duplicate. The person carrying out the assays was completely blinded to the clinical information.

### Blood monocyte subset redistribution

The samples were further stained with anti-CD16-PE and anti-CD14-FITC mAbs (BD Biosciences, San Jose, CA, USA) to identify monocyte subsets. Class-matched isotype immunoglobulin negative control mAbs (BD Biosciences, San Jose, CA, USA) was added to separate tubes for the same samples to detect nonspecific binding. The samples were incubated away from light at room temperature for 15 to 30 min. The samples were then incubated with hemolysin until all red blood cells cracked. The plates were washed with 500 μL of PBS and centrifuged at 1400 r/min for 5 min. After the supernatant was removed, the cells were washed again, resuspended in paraformaldehyde, and analyzed by flow cytometry. FACS Calibur software (Beckman–Coulter, USA) was used for cytometric analysis. The analyses were done in duplicate and the technician was blinded to clinical data.

### Delirium evaluation

POD was assessed through Nu-DESC scoring at the same time on the first to third day postoperatively. Five parameters were captured; each with a subscore of 0–2 points (Table 1). A total of ≥ 2 points indicated the presence of delirium [16].

**Table 1:** Nursing delirium screening scale

Symptom	Symptom rating		
1 Disorientation	0	1	2
Verbal or behavioral manifestation of not being oriented to time or place or misperceiving persons in the environment			
2 Inappropriate behavior	0	1	2
Behavior inappropriate to place and/or for the person; e.g., pulling at tubes or dressings, attempting to get out of bed when contraindicated, and the like			
3 Inappropriate communication	0	1	2
Communication inappropriate to place and/or for the person; e.g., in-coherence, non-communicativeness, nonsensical or unintelligible speech			
4 Illusions/Hallucinations	0	1	2
Seeing or hearing things that do not exist; distortions of visual objects			
5 Psychomotor retardation	0	1	2
Delayed responsiveness, few or no spontaneous actions/words; e.g., when the patient is prodded, the reaction is deferred and/or the patient is unarousable			

### Statistical analysis

All data were analyzed by SPSS (version 21.0 for Windows, SPSS Inc., Chicago, IL, USA). Categorical variables were analyzed through *χ* ^2^ test or Fisher’s exact test and presented as numbers and percentages. Continuous variables were tested with Student’s *t* test for normal distribution or Mann–Whitney *U* test for skewed distribution and reported as mean ± standard deviation.

Mediation can be demonstrated using four steps: (1) the effect of the independent variable (HS) on the dependent variable (delirium) must be significant, (2) the path from the independent variable (HS) to the mediator (CD14 + CD16+ monocytes) must be significant, (3) the path from the mediator (CD14 + CD16+ monocytes) to the dependent variable (delirium) must be significant, and (4) the independent variable (HS) has a reduced or no effect on the dependent variable (delirium) after adjustment for the mediator (CD14 + CD16+ monocytes) [17].

With steps 3 and 4 tested simultaneously, three mixed-effect logistic regression models were used to test (1) the association between prophylactic HS and delirium the following day, (2) the association between CD14 + CD16+ monocytes and delirium, and (3) the association between prophylactic HS and delirium controlling for CD14 + CD16+ monocytes [18]. Another multivariate logistic regression model was employed to determine the association between cytokines and delirium. Associations were assessed with 95% CI and considered significant at *P* values less than 0.05.

## Results

A total of 120 patients who underwent surgery for a femoral neck fracture were enrolled in the study and assigned into two groups. Table 2 shows the main demographic, chronic medical conditions, and the incidence of delirium. No statistically significant differences occurred in the age, gender, BMI, and comorbidities (all *P* > 0.05). Cytokines and pro-inflammatory monocytes were significant between the two groups (*P* < 0.01). The incidence of delirium was statistically significantly lower in HS group than in NS group (*P* = 0.001).

**Table 2:** Baseline demographic variables, subject characteristics, and prevalence of postoperative delirium

Variable	Hypertonic saline (*n* = 60)	Normal saline (*n* = 60)	*P* value
Demographic and clinical characteristics			
Age (years)	76.6 ± 5.8	75.6 ± 5.6	0.382
Sex, female (%)	31 (52)	27 (45)	0.534
BMI (kg/m^2^)	28.7 ± 3.4	28.5 ± 3.3	0.845
Education (years)	9.2 ± 3.3	9.6 ± 3.3	0.492
ASA score of 2, *n* (%)	38 (63.3)	35 (58.3)	0.315
MMSE (point)	25.7 ± 1.4	25.5 ± 1.3	0.343
Duration of anesthesia (min)	98.5 ± 12.3	102.2 ± 13.3	0.113
Frequency of comorbidities:			
Coronary artery disease, *n* (%)	14 (23.3)	18 (30)	0.682
Diabetes, *n* (%)	15 (25.0)	10 (16.7)	0.261
Hypertension, *n* (%)	47 (78.3)	50 (83.3)	0.484
Cerebral infarction, *n* (%)	14 (23.3)	19 (31.7)	0.307
Cytokines			
IL-1β (pg/ml)	1.33 ± 0.17	1.75 ± 0.15	*0.000*
IL-6 (pg/ml)	91.03 ± 9.32	121.44 ± 9.58	*0.000*
IL-10 (pg/ml)	6.89 ± 1.78	5.45 ± 1.68	*0.000*
TNF-α (pg/ml)	20.64 ± 3.78	44.03 ± 3.52	*0.000*
S100β (ng/mL)	0.26 ± 0.09	0.34 ± 0.11	*0.000*
CD14 + CD16+ monocytes (%)	19.70 ± 4.57	35.75 ± 4.31	*0.000*
Prevalence of delirium, *n* (%)	7 (11.7)	23 (38.3)	*0.001*

*BMI* body mass index, *ASA* American Society of Anesthesiologists, *MMSE* mini-mental state examination. Significant results are italicized.

### Association between prophylactic hypertonic saline and delirium

The independent variable was set as HS (yes or no) administered before anesthesia, and the dependent variable was set as the presence of delirium the following day in the multivariate models to evaluate the effect of HS on elderly patients with POD. In the multivariate analysis model, age, sex, ASA class, BMI, duration of anesthesia, and comorbidities were compared between the non-delirium and total delirium groups. Analysis using logistic regression models indicated HS administration before anesthesia was associated with a low risk of POD (odds ratio [OR], 0.13; 95% CI, 0.04 to 0.41; *P* = 0.001) (Table 3). Furthermore, age, sex, and cerebral infarction remained significant (all *P* < 0.05). However, the duration of anesthesia, ASA class, MMSE, and other comorbidities were statistically meaningless in relation to delirium.

**Table 3:** Random-effect logistic regression model showing associations between hypertonic saline and delirium

	OR	95% CI	*P* value
Hypertonic saline	0.13	0.04 to 0.41	*.001*
Age, per year	1.16	1.05 to 1.28	*.005*
Sex, women versus men	0.30	0.10 to 0.89	*.030*
ASA, II versus III	1.41	0.49 to 4.09	.525
MMSE, per point	0.89	0.60 to 1.31	.540
Hypertension, yes versus no	1.33	0.37 to 4.79	.664
Diabetes, yes versus no	1.81	0.54 to 6.09	.341
Coronary artery disease, yes versus no	0.90	0.26 to 3.19	.873
Cerebral infarction, yes versus no	3.76	1.28 to 11.07	**.** *016*
Duration of anesthesia, < 97 min versus ≥ 97 min	1.92	0.63 to 5.83	.252

*ASA* American Society of Anesthesiologists, *MMSE* mini-mental state examination, *CI* confidence interval, *OR* odds ratio The receiver operating characteristic (ROC) curve was calculated to evaluate the cut-off point of the duration of anesthesia. The patients were divided into two groups based on the cut-off point. Significant results are italicized

### Association between prophylactic HS and CD14 + CD16+ monocytes

**Fig. 1:**
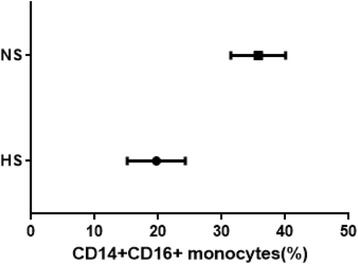
Relationship between the levels of CD14 + CD16+ monocytes according to prophylactic hypertonic saline (HS) before anesthesia. Each point shows the average CD14 + CD16+ monocytes (%) in each group along with standard deviation

HS was associated with low levels of CD14 + CD16+ monocytes (Fig. 1). The association between HS and CD14 + CD16+ monocytes was assessed using a linear mixed-effect model. The independent variable was set as HS, and CD14 + CD16+ monocyte level was set as the dependent variable on the first day after the surgery. Linear regression analysis demonstrated the significant association between monocyte level and HS (β = − 0.61; 95% CI, − 0.74 to − 0.48; *P* = 0.000) (Table 4). The duration of anesthesia was associated with the level of CD14 + CD16+ monocytes (*P* = 0.030).

**Table 4:** Linear regression model showing associations between hypertonic saline and CD14 + CD16+ monocytes

	β	95% CI	*P* value
Hypertonic saline	− 0.61	− 0.74 to − 0.48	*.000*
Age, per year	0.00	− 0.00 to 0.02	.160
Sex, women versus men	− 0.07	− 0.20 to 0.07	.327
ASA, II versus III	0.05	− 0.09 to 0.18	.486
MMSE, per point	− 0.03	− 0.08 to 0.01	.166
Hypertension, yes versus no	−0.06	− 0.22 to 0.10	.464
Diabetes, yes versus no	0.04	− 0.11 to 0.20	.588
Coronary artery disease, yes versus no	− 0.09	− 0.25 to 0.06	.242
Cerebral infarction, yes versus no	0.05	− 0.09 to 0.19	.456
Duration of anesthesia, < 97 min versus ≥ 97 min	0.15	0.01 to 0.28	*.030*

*ASA* American Society of Anesthesiologists, *MMSE* mini-mental state examination, *CI* confidence interval, *OR* odds ratio. The ROC curve was calculated to evaluate the cut-off point of the duration of anesthesia. The patients were divided into two groups based on the cut-off point. Significant results are italicized

### Association between prophylactic HS and delirium after adjustment for CD14 + CD16+ monocytes

HS and CD14 + CD16+ monocyte levels were set as the independent variables, and delirium was set as the dependent variable. The effect of HS on the probability of delirium decreased (odds ratio [OR], 0.86; 95% CI, 0.14 to 5.33; *P* = 0.874) when accounting for CD14 + CD16+ monocytes (Table 5). Delirium remained significantly related to CD14 + CD16+ monocytes (odds ratio [OR], 14.51; 95% CI, 2.41 to 87.38; *P* = 0.004) (Table 5). Analysis using the multivariable logistic regression model indicated that age, sex, and cerebral infarction remained associated with the development of postoperative delirium after adjusting for other important preoperative factors (all *P* < 0.05).

**Table 5:** Random-effect logistic regression model showing the relationship between hypertonic saline and delirium, adjusted by CD14 + CD16+ monocytes

	OR	95% CI	*P* value
Hypertonic saline	0.86	0.14 to 5.33	0.874
CD14 + CD16+ monocytes, ≤ 34.40% versus > 34.40%	14.51	2.41 to 87.38	*0.004*
Age, per year	1.16	1.04 to 1.29	*0.009*
Sex, women versus men	0.31	0.09 to 0.99	*0.048*
ASA, II versus III	1.40	0.45 to 4.37	0.562
MMSE, per point	0.98	0.65 to 1.48	0.910
Hypertension, yes versus no	1.49	0.38 to 5.94	0.569
Diabetes, yes versus no	1.64	0.44 to 6.08	0.461
Coronary artery disease, yes versus no	1.34	0.35 to 5.09	0.669
Cerebral infarction, yes versus no	3.73	1.16 to 11.97	*0.027*
Duration of anesthesia, < 97 min versus > 97 min	1.27	0.38 to 4.27	0.694

*ASA* American Society of Anesthesiologists, *MMSE* mini-mental state examination, *CI* confidence interval, *OR* odds ratio. The ROC curve was calculated to evaluate the cut-off point of CD14 + CD16+ monocytes and the duration of anesthesia. The patients were divided into two groups based on the cut-off point. Significant results are italicized

**Fig. 2:**
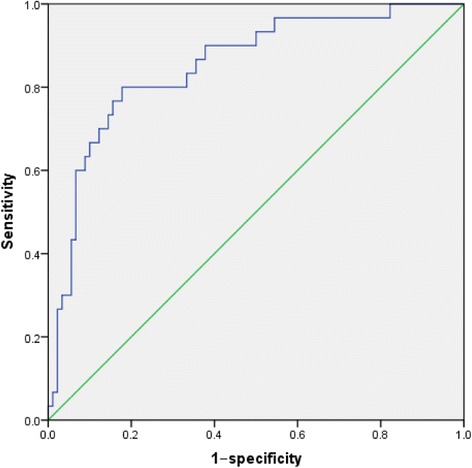
Analysis of the receiver operating characteristic for the predictive value of delirium

Cytokines and perioperative factors were identified using a stepwise logistic regression model and the occurrence of delirium as the dependent variable. TNF-α remained significant after adjusting for other risk factors in the multivariate models (odds ratio [OR], 1.10; 95% CI, 1.05 to 1.16; *P* = 0.000) (Table 6). The positive predictive ability of the multivariate logistic regression model tested by ROC analysis showed an area under the curve of 0.851 (95% CI, 0.77 to 0.93) (Fig. 2).

**Table 6:** Stepwise logistic regression model showing the relationship between cytokines and delirium

	OR	95% CI	*P* value
TNF-α (pg/ml)	1.1	1.05 to 1.16	0.000
Age, per year	1.15	1.04 to 1.27	0.006
Sex, women versus men	0.27	0.09 to 0.77	0.014
Cerebral infarction, yes versus no	2.98	1.05 to 8.42	0.039

## Discussion

In this study, HS can reduce the development of postoperative delirium in geriatric patients after adjusting for age, sex, and comorbidities. The underlying mechanism may be related to the inhibition of the secretion of inflammatory factors by monocytes. The anti-inflammatory action may form a part of the basis of HS–delirium relationship, and this finding confirms the neuroinflammatory hypothesis of delirium.

Treatment with HS could be responsible for a stretching relief that could influence natriuretic and immuno-inflammatory markers [19]. Liu et al. [20] also confirmed the function of HS in improving inflammation. In the present study, we investigated the relationship between HS and postoperative delirium. Systemic inflammation is a prominent feature of numerous medical and surgical conditions associated with delirium, particularly when these conditions involve tissue destruction or infection [6]. A strong association was found between the elevated levels of inflammation biomarkers and the risk for developing delirium [8, 11, 21]. We primarily determined the correlation of HS and postoperative delirium and identified whether or not their relationship was statistically significant. The results provide proof and principle for large trials to further study clinical outcomes.

The inflammatory and metabolic effect of saline overload correction in treatment of cirrhosis complications suggested a possible role of inflammatory and metabolic-nutritional variables as severity markers in these patients [22]. Moreover, several investigations have characterized HS in terms of its specific immune-modulatory qualities, such as monocyte subset redistribution and cytokines production [20, 23]. The activity of proinflammatory cytokines is thought to be a critical link between cognitive impairments and POD pathogenesis [24]. In addition, HS inhibits pro-inflammatory cytokine production but stimulates anti-inflammatory cytokine production by monocytes/macrophages [12]. Mahnaz et al. [25] conducted a clinical trial involving 40 patients who underwent elective coronary artery bypass grafting (CABG) surgery; those who received HS exhibited lower level of IL-6 and higher level of IL-10 than those given with NS 48 h after the surgery. In addition, compared with treatment with furosemide alone, treatment with furosemide/HSS resulted in a significant lowering of plasma levels of atrial natriuretic peptide (ANP), BNP, TNF-α, IL-1, and IL-6 [26]. Thus, we investigated the plasma levels of cytokines and monocytes.

Of all proinflammatory cytokines, TNF-α mediating immune responses is regarded as the initiating medium of systemic inflammatory responses. TNF-α can enhance the adhesive capacity of neutrophils and monocytes; induce the release of IL-1β, IL-6, IL-8, and adhesion molecules; and eventually exert a cascade amplification effect on inflammation [27]. Surgery leads to the production of TNF-α, which subsequently disrupts the blood brain barrier and causes the infiltration of inflammatory macrophages in the brain parenchyma, specifically in the hippocampus [27]. IL-1β plays a pivotal role in surgery-induced cognitive dysfunction. Memory impairment caused by surgery intervention is related to the increased levels of plasma cytokines and the hippocampal neuron IL-1β pathway [28]. IL-6 is a critical cytokine connecting innate and acquired immunity, and is vital for induction of both peripheral and central defense against injury and inflammation [29]. In addition, IL-6 facilitates the effect of IL-1β in mediating inflammation and causing hippocampal-dependent memory impairment [30]. Thus, the level of pro-inflammatory cytokines, including IL-1β, IL-6, TNF-α, and anti-inflammatory IL-10, may be related to the pathophysiology of POD and can be determined by ELISA. Furthermore, cerebral or extra-cerebral neuron damage can increase the serum level of S100β, which is a response of the brain to proinflammatory cytokines [31]. In the present study, preconditioning HS decreased the levels of IL-1β, IL-6, TNF-α, and S100β and increased the level of IL-10 compared with those in the NS group. Hence, HS significantly influenced the secretion of inflammatory and anti-inflammatory cytokines. Similarly, another study reported that HS effectively inhibits pro-inflammatory cytokines and enhances the production of counter-inflammatory cytokines [32]. It is also consistent with the previous study, in which higher S100β levels were found in patients with delirium than in patients without delirium [31].

An animal study confirmed the negative effect of elevated levels of pro-inflammatory cytokines in hippocampus [33]. We also tested the association between cytokines and delirium. Multivariate logistic regression analysis demonstrated the significant association of delirium with TNF-α and the receiver operating characteristics analysis for the predictive value of delirium showed an area under the curve of 0.851. Our findings support that acute systemic inflammatory TNF-α can sufficiently induce acute dysfunction in a cognitive task reliant on attentional and working memory function; two key cognitive domains were impaired in delirium [34]. Elevated TNF-α level could alter human cognitive and affective behavior. However, previous studies have had conflicting results that TNF-α did not associate with delirium [35]. Further studies are needed to confirm these imparities.

TNF-α and IL-10 are the main inflammatory cytokines and are mainly produced by monocytes and macrophages [36, 37]. Monocytes positively express HLA-DR and CD11b, and differentially express CD14 and CD16 in humans [38]. Classical monocytes (CD14++CD16–), which comprise peripheral blood monocytes, can migrate to injury and infection sites, where they differentiate into inflammatory macrophages. The subset of intermediate monocytes (CD14 + CD16+) generally exhibits inflammatory characteristics [12]. The increase in the levels of monocytes (CD14 + CD16+) indicates the presence of systemic inflammatory responses. Rizoli et al. [12] reported that HS can inhibit inflammation by suppressing CD14 + CD16+ monocytes. Using flow cytometry analysis, we found the significantly lower level of CD14 + CD16+ monocytes in patients of the HS group than those of the NS group. In addition, logistic regression analysis demonstrated the strong association between CD14 + CD16+ monocytes and delirium. These results indicate that surgery caused the selective expansion of pro-inflammatory CD14 + CD16+ monocytes in peripheral blood, and the probability of delirium occurrence is due to CD14 + CD16+ monocytes redistribution. Hence, HS can reduce the occurrence of POD, which might inhibit CD14 + CD16+ monocytes, resulting in reduced release of inflammatory factors.

The observed effect after HS administration suggests a biologically plausible causal pathway, where the reduction in systemic inflammation mediates the relationship of HS and delirium. In the analysis, the criteria for mediation were met. Prophylactic HS (independent variable) was associated with delirium (dependent variable) and with CD14 + CD16+ monocytes (mediating variable). CD14 + CD16+ monocytes were associated with delirium. Adjusting the association between prophylactic HS and delirium by CD14 + CD16+ monocytes showed a reduction in effect size. Thus, prophylactic HS could be associated with a low risk of delirium in geriatric patients who underwent hip surgery.

In this study, we identified TNF-α, age, sex, and cerebral infarction as independent risk factors for the occurrence of POD. This finding is consistent with previous reports that age and male sex are the major risk factors for the development of delirium [39]. Age and sex, which cannot be modified through medical intervention, are demographic risk factors associated with delirium. Changes in the neurons or neurotransmitters in the brain can lead to an increased risk of cognitive disruption in patients with cerebral infarction. The relationship between acute stroke and delirium has also been well-established. Delirium may be a non-specific finding of acute stroke and may occur in the brain area affected by stroke [40]. Tuttolomondo et al. [41] suggested a possible role for a T-cell component in acute ischemic stroke clinical setting showing a different peripheral frequency of CD4 + CD28− cells in relation of subtypes of stroke and the role of CD4 + CD28− subset could represent a natural extension of cytokine, selectins, and adhesion molecule activation [42]. In addition, WBC could represent a marker of inflammatory process that is well recognized to have a role in the pathogenesis of the ischemic neuronal damage [43]. CD4 + CD28− cells and WBC may be involved in POD induced by stroke.

This study found that the incidence of delirium in elderly patients who underwent hip replacement was about 25%. This finding is consistent with the results of previous studies [4]. In aged rats, peripheral stimulation induces high levels of cytokines (IL-1β, IL-6, and TNF-α) in the hippocampus and retains them for a long time [7]. The duration of delirium is an independent risk factor of postoperative cognitive dysfunction (POCD) in aged patients after surgery [10]. POCD is characterized by persistent cognitive difficulties, including memory and attention problems and executive dysfunction, and commonly occurs after the surgery. We previously demonstrated that 7.5% HS can improve cognitive function after the surgery of aged rats, and the underlying mechanism may be related to the inhibition of hippocampal neuron apoptosis [44]. Pre-clinical experiments confirmed the relationship between the inflammation of hippocampus neurons and POCD [45]. TNF-α exerts robust acute effects on brain function in the degenerating brain and is highly relevant for the illness-induced exacerbations of brain dysfunction, including depression, delirium, and POCD [46]. The effect of HS on reducing hippocampal neuron apoptosis by reducing neuroinflammation to improve POCD remains to be discussed.

Patients who have pre-existing cognitive impairment are more likely to show cognitive impairment [47]. Thus, we tested MMSE with patients before the surgery to rule out confounding factors. The current research exhibits certain limitations. The number of patients is relatively small because of the strict exclusion criteria. We only enrolled 120 patients, and the CI was insufficiently precise. Despite the multiple adjustments conducted, residual confounding remains a possibility. Time between hip fracture and subsequent surgery, preoperative medications, pain, and use of opioids and other drugs postoperatively may as well affect cognitive status. The observation time is short. A long observation period may be important in determining the relationship between the subsets of delirium and the level of inflammatory factors. Prolonging the observation time might be helpful in guiding the management of delirious patients. The limitation of cognitive assessment in geriatric patients is recognized. The Nu-DESC test is designed to be administered by a bedside nurse based on clinical observations in their routine practice. This test has very high sensitivity and low specificity [16]. At present, confusion assessment method is the most widely used tool for highly sensitive and specific screening of delirium. As well as, we merely collected plasma to detect the inflammation which cannot abundantly represent its level of cerebrospinal fluid. Increased systemic, serum cytokine levels may precede an increased neuroinflammatory response associated with delirium [48]. The peripheral inflammation may have altered central nervous system cytokine profiles through several channels reportedly [49–51].

## Conclusion

Prophylactic HS exerts beneficial effect on delirium in geriatric population, and such effect may be related to inhibition of monocytes. However, the favorable effects of HS on improving long-term treatment outcomes remain unknown. Further work should be conducted to elucidate the mechanisms by which HS can intervene during the development of delirium.

## Abbreviations

ASA: American society of anesthesiologists
BMI: Body mass index
CABG: Coronary artery bypass grafting
CI: Confidence interval
HS: Hypertonic saline
IL-1β: Interleukin-1β
MMSE: Mini-mental state examination
NS: Normal saline
Nu-DESC: Nursing delirium screening scale
OR: Odds ratio
POCD: Postoperative cognitive dysfunction
POD: Postoperative delirium
ROC: Receiver operating characteristic
TNF-α: Tumor necrosis factor alpha
